# Inviting ecosystems into the exposome framework

**DOI:** 10.1093/exposome/osag007

**Published:** 2026-02-20

**Authors:** Claire Villette, Gary W Miller, Jean Sibilia, Dimitri Heintz

**Affiliations:** Université de Strasbourg, CNRS, IPHC UMR7178, Strasbourg, France; Department of Environmental Health Sciences, Mailman School of Public Health, Columbia University, New York, NY, USA; Rheumatology Department, Centre National de Référence des Maladies Auto-immunes Systémiques Rares RESO, Strasbourg University Hospital, Strasbourg, France; Université de Strasbourg, CNRS, IPHC UMR7178, Strasbourg, France

**Keywords:** ecosystem health, exposome, one health, boomerang effect, nature-based solutions, mirror research, interdependency

## Abstract

Historically and willingly, the exposome framework focuses on human health. Ecosystems are considered only partially and are always envisioned as part of the exposures. As knowledge expands on the human exposome, human-nature relationships and interdependency are becoming increasingly obvious, with an impact of nature on human health and well-being. Including ecosystems into the exposome framework would help to identify shared exposures, boomerang effects, specific adaptations occurring in other living beings, nature-based solutions to mitigate identified problematics linked to human health, and maintain a habitable environment for all living beings.

## Introduction

Since the word exposome was proposed by Christopher Wild in 2005 as a complement to the genome,^1^ incredible advances have been achieved in the area of human health. The scientific community accepted the idea that the genome by itself was not sufficient to explain the occurrence of diseases in humans. Taking into account the diversity of exposures throughout the entire lifespan and the biological response to these exposures was a great challenge embraced by the community. The outlines of the exposome framework have been further refined by several investigators^2-5^ and the topic has been the focus of numerous meetings, workshops, and journals. More recently the Banbury Exposomics Consortium^6^^,^^7^ proposed a unified definition to advance exposomics in biomedical research. The establishment of best practices and harmonization of data collection are on the way to help further interactions and discoveries.^8^ Searching for the causes of diseases to allow prevention is the key in future medical care. Indeed, governments and doctors are already warning that tomorrow’s medicine will not be able to bear the cost of healthcare for all in the future.

**Figure 1.**
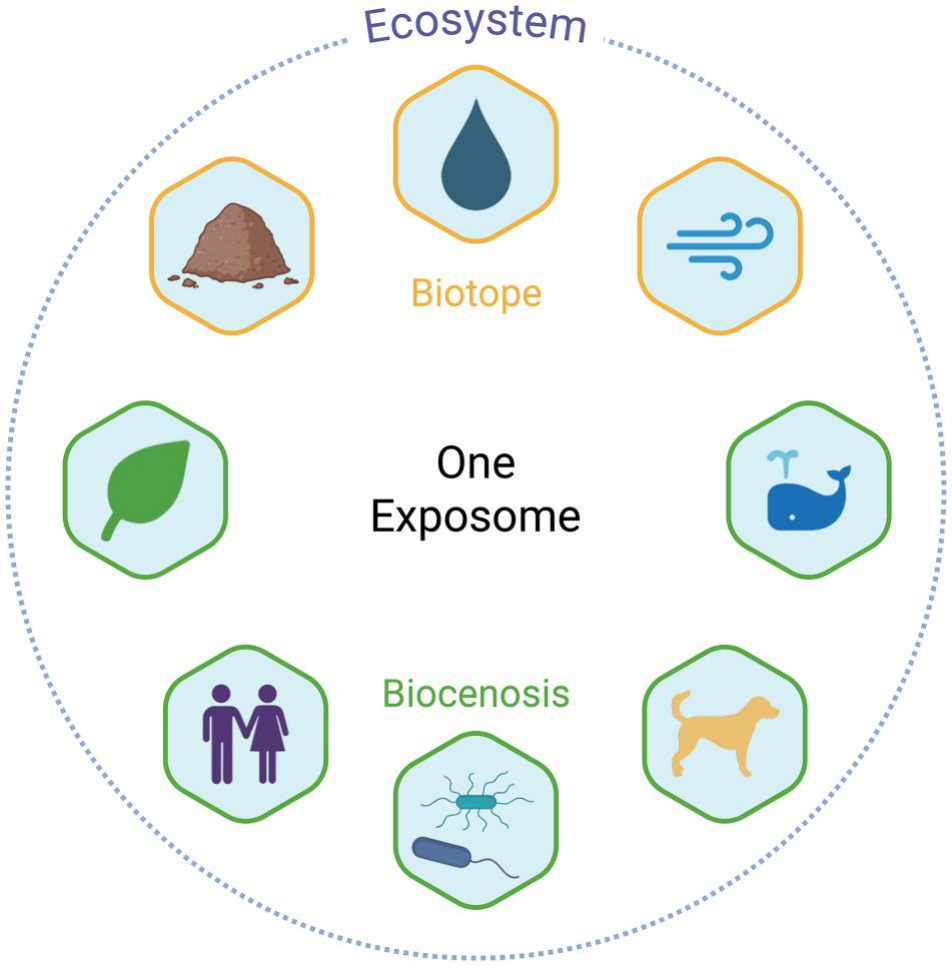
Ecosystems, composed of biotope and biocenosis (including humans), share the same exposome. The composition of the various biotopes and biocenosis exist as complex networks with numerous multi-direction interactions. From a practical perspective, it may be necessary to study various subsets of the complex uber-network, but one should consider the interconnectedness of the components. Created in BioRender. Heintz, D. (2025) https://BioRender.com/x73o6i4.

Now that it is widely admitted that environmental exposures are key players in human health, we have to assemble the next piece of the ever-growing puzzle. Until now, sapiocentrism was the rule of thumb but humanity is part of nature as we belong to the animal kingdom that inhabits the Earth. As such, we suffer from a diseased environment which directly impacts our lives: environmental disasters such as floods and heat waves, loss of biodiversity that impact agriculture and human well-being,^9^ or disease outbreaks resulting from wild species living in close proximity to humans due to habitat loss,^10^ as recently reminded by the COVID-19 pandemics. This does not even take into consideration the effects of poor air or water quality or the poor nutritional content of our daily food. Recently, scientists asked for a redefinition of human health to take into account environmental exposures.^11^ We should also start understanding the health status of ecosystems, to avoid living in a poor environment, displaying increasing climatic and biodiversity challenges, which in turn makes us sick. To this end, we propose to invite ecosystems into the exposome framework with a One Exposome approach, as a parallel to the One Health approach, which recognizes interconnections between the health of humans, animals and their environment (along with EcoHealth and Planetary Health, which take different entry points to the question^12^). We propose to consider humans, animals and the environment as a single continuum in which exposures are shared (Figure 1). The exposome and One Health frameworks were recently discussed to have common interests but also specific focus.^13^^,^^14^ Here, we wish to emphasize that all the entities of ecosystems share part of their general external exposome, which might alter the health status of living beings.

**Figure 2.**
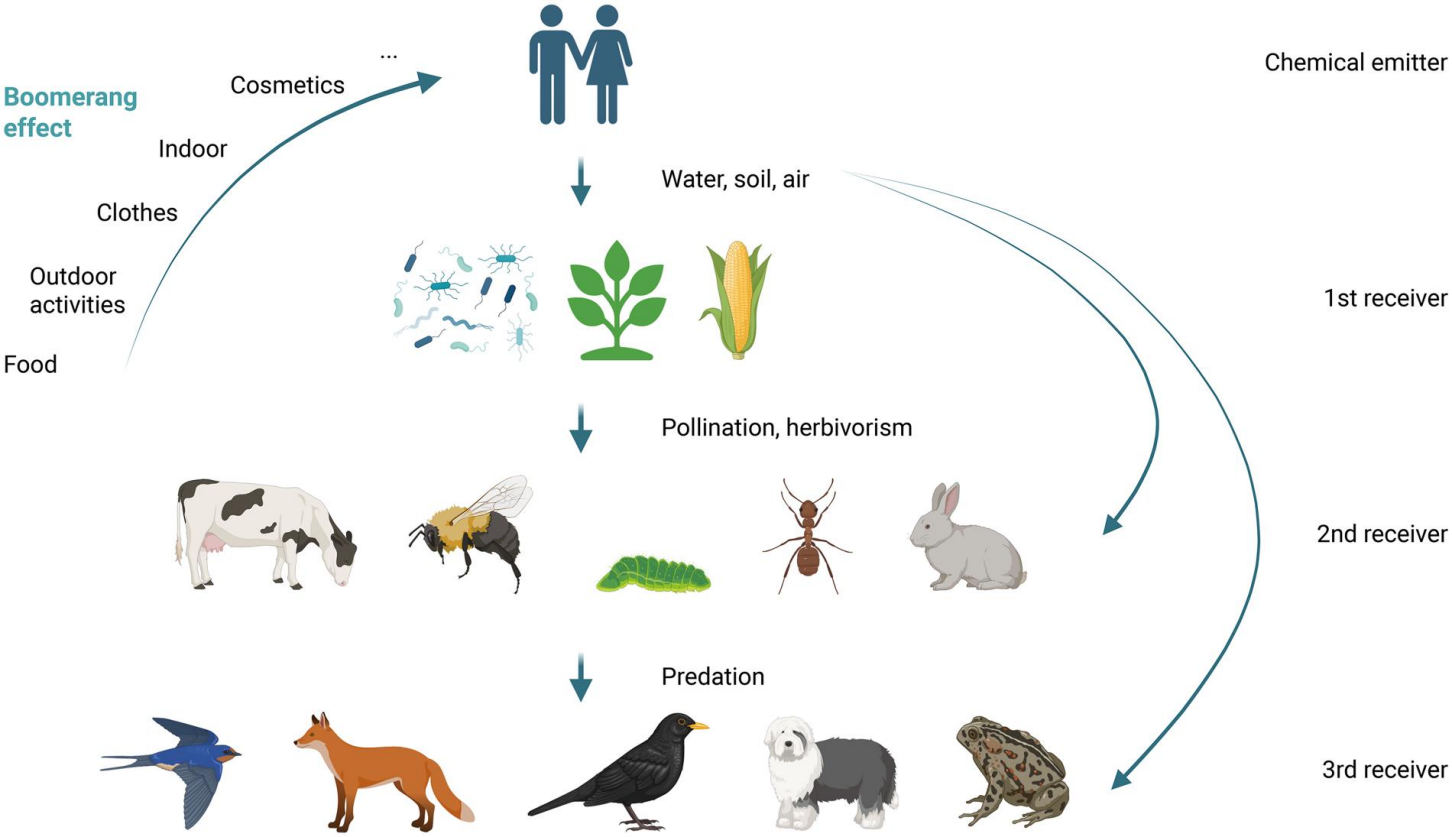
The boomerang effect. An example of the boomerang effect illustrated with chemicals released by anthropogenic activities (micropollutants), spreading through trophic networks and coming back to humans. Many human medications are excreted through urine and make their way into the water systems where they are absorbed or consumed by the 1st, 2nd, or 3rd receiver. Alternatively, the 2nd or 3rd receiver may be exposed by ingestion of the 1st receiver leading to the classic bioaccumulation seen throughout nature. Created in BioRender. Heintz, D. (2025) https://BioRender.com/93lia25.

Ecosystems are composed of the biotope and the biocenosis. The biocenosis consists of all the living organisms, while the biotope provides the habitat for life.^10^ Biotope and biocenosis are widely variable depending on their location on Earth, with some species specific to a single biotope (ecological niche), while others might develop in a variety of environmental contexts. Interactions are key to viable ecosystems, with metabolic and energetic exchanges. This also means interconnections and shared environmental exposures between organisms of the same ecosystem, including humans (Figure 1). In this context, processes like bioaccumulation of anthropogenic pollutants might occur throughout trophic networks or water cycles. We need a stronger understanding of these interactions to mitigate environmental constraints that affect ecosystems, and might affect human health through a boomerang effect (Figure 2). Indeed, anthropogenic pollutants mostly contaminate air, soil and water directly. Then, wild plants, microbes or crops growing from contaminated soil or water accumulate pollutants as first receivers. Plants are further eaten or pollinated by herbivores and pollinators (second receivers), which themselves have predators (third receivers). Following trophic networks, anthropogenic pollutants might disperse and/or accumulate. Then, humans can come again into contact with contaminated water, soil, craft products (clothes, cosmetics) or eat food from contaminated crops or animals. This is what we call the boomerang effect, as pollutants emitted by humans come back to their daily lives as new exposures.

It should be noted that the idea of linking ecosystems with the exposome is not new. Indeed, the late Paul Lioy suggested the term eco-exposome as a framing for this interaction. This terminology was used in the U.S. National Research Council Report on Exposure Science in the 21^st^ Century^15^ as a way to address exposures that derive from outside of the body (similar to what Christopher Wild described as the general external exposome). Since that time, these external factors are clearly included in the definition of the exposome, eg, the Banbury Definition.^7^ At the same time, it appeared that the report from Dr Lioy was more focused on exposures that come from the ecosystem and not the exposome of the ecosystem itself, namely the internal and external exposomes for living beings (biocenosis), and the external exposome for biotopes, which might be shared and exchanged between entities. A recent initiative tended to record data from the environment to include it into the exposome framework.^16^ These data mainly represent abiotic factors (air, water, soil) obtained from environmental agencies, with the ultimate goal to be linked to human health risk. As noted above, most research on the exposome has continued to be human/sapiocentric, thus requiring the distinction introduced in this commentary.

## Health status and exposome of ecosystems

It might be difficult to define the health status of ecosystems and the causative exposures. Climate and biodiversity challenges are increasingly documented, supporting the idea of a “non-healthy” ecosystem. Aldo Leopold studied land health resilience by observing its self-renewal capacity.^17^ At that time (1949), he already identified loss of biodiversity and invasive species as two types of sickness for the environment. The magnitude of climatic disorders and their frequency could now be added to the list. Today, to define a good health status for ecosystems, one might take into consideration some sort of stability of the environment over time (avoiding catastrophic events) and preserving conditions that are compatible with life as we know it (avoid heating temperatures, ocean acidification, habitat loss, and species extinctions). This can be summed up by language used in the Washington, D.C. Declaration on the Human Exposome (https://exposomemoonshot.org/washington-d-c-declaration-on-the-human-exposome/) recently signed at the Exposome Moonshot Forum (2025): “We’ve launched a mission not just to map what makes us sick, but to protect what keeps us well.” Maintaining biodiversity and ecosystem services would be the golden aim. To this end, ensuring the good quality of air, water and soil is mandatory. Avoiding catastrophic climatic events is also part of the challenge, as the weather impacts the health of living beings.^18^ However, we must put the concept of ecosystem health into perspective, as this view is centered on how humans perceive nature. The good health of an ecosystem reflects what we want to see in a healthy ecosystem, but it may be completely different from a microorganism point of view. For example, one can imagine a beautiful meadow with fruit trees, hedges, rich soil and abundant biodiversity. This would be considered a healthy ecosystem from the human point of view. Then, imagine that an industrial disaster occurs, resulting in heavy metal pollution spreading across the beautiful meadow. One would then say that this now polluted ecosystem is in poor condition and therefore in poor health, and rightly so. These polluted conditions might be deleterious for the current biocenosis, which might perish. But for soil microbes, for example, which specialize in using heavy metals for their metabolism,^19^^,^^20^ this polluted meadow is very good news. These microbes, which could not live there before the pollution, can now do so. For this specialized microbiota, the polluted ecosystem is ultimately habitable. Therefore, restorative processes occur in this polluted biotope, and ecosystems have to adapt with the reestablishment of an ecological balance, which occurs through the rise of a new biocenosis (eg, adapted microbiota). This new biocenosis might only be temporary, allowing the restoration of the original conditions, depicting ecosystem resilience. This is to remind that our focus is not on specific or specialized organisms, but rather on the health and stability of existing whole ecosystems, which include the biotope and the original biocenosis. In healthy ecosystems, viable metabolic and energetic exchanges occur between organisms. Then, a healthy ecosystem would be defined as an ecosystem able to achieve adaptation, maintain ecological balance and equilibrium through feedback loops, show self-renewal capacity, therefore depicting resilience capabilities when faced to “traumatic” events. As Hippocrates already stated, health is achieved through the equilibrium between an organism and its environment.^21^

Medical analogies between human diseases and ecological issues have already been used in the past by considering ecological issues as diseases, finding their causes and mitigating the imbalance to recover a controlled equilibrium or homeostatic state.^22^ This applies mainly to the biocenosis consortium in ecosystems, as the biotope is not living. Profound changes in landscape and environment can cause solastalgia (sometimes called eco-anxiety), a mental distress measured in humans when their place of residence is threatened or transformed by anthropogenic activities.^23^^,^^24^ The transformations leads to a new equilibrium, the place is not recognized anymore as the comfortable home it used to be, and homesickness appears even though people are still at home. Maintaining habitable landscapes from the biological point of view, but also mentally acceptable environments is part of the current urban strategies and projects over the world (eg, the Urbact project which aims at adapting cities to fit in the One Health framework, https://urbact.eu). For example, the amount of bird species in the environment is positively correlated to human well-being,^9^ which also benefits more globally to biodiversity and therefore ecosystems health (eg, birds eat mosquitoes and avoid the spread of vector-borne diseases). Using nature-based solutions as bioremediation can help mitigate micropollutants, taking advantage of the powerful work made by microbes^25^ and plants^26^ to maintain habitable ecosystems. These are two examples of the numerous services provided by ecosystems, which improve human health. A nice illustration is the phytoremediation and pollution mitigation operated by plants in constructed wetlands, where part of the pollutants from domestic wastewater are sequestered in plant tissues and/or degraded by the plant.^26^^,^^27^ Then, polluted plant cuttings can be used to produce fertilizer (usable in agriculture), through the development of a microbial consortium which consumes the polluted plant tissues and remediates the remaining micropollutants, cost-free, with no energy input and low CO_2_ emissions.^28^ Studying ecosystems exposome and finding solutions to keep it in a good health status is a way to take care of human health and maintain a favorable exposome. Additionally, the history of biological sciences suggests that we must also learn to observe non-animals, particularly plants, preparing us for the unusual, the counterintuitive and, therefore, the innovation.

## Studying the animal exposome might benefit to human health

In close proximity to humans are domestic animals, which share our lifestyles: they live in enclosed areas, away from their original natural environment. They eat industrialized food; have low levels of physical activity, and a smaller roaming range. Therefore, they suffer the same conditions as humans, sometimes correlated with industrialized food and low physical activity: cancer, obesity, cardiac disorders, senility or impaired fertility. They even share an illness that one might consider exclusive to human, namely nervous depression.^29^ Domestic animals might be considered as sentinel species to guide us towards human health issues, social welfare and maybe remedies.^30^^,^^31^

Wild animals share part of their exposome with humans: environment, weather, air/soil/water quality, climatic disorders, anthropogenic pollutants etc, Certain species use man-made materials to make their shells^32^^,^^33^ or nests^34^ and therefore benefit from humans, although the costs of such use is not yet completely known in terms of health (exposure to microplastics). Others suffer from plasticosis after ingesting plastics, even at very young ages.^35^ The diversity of biological responses to similar environmental constraints in the animal kingdom is incredibly interesting to understand the exposome. This diversity echoes the individual variability observed in humans, which brought us to precision and personalized medicine, as the same causes do not always produce the same effects in distinct persons. There is an individual variability factor in the nature of the biological response after an exposure to exogenous chemicals (xenobiotics). For example, chemicals acting as endocrine disruptors are very difficult to detect using conventional toxicology tests, and they might affect some individuals but not all. Not every xenobiotic affects each and every one of us, but their diversity is such that each and every one of us is affected by at least one of them.

Human health might also benefit from discoveries made on animal species that display incredible traits. For example, some long-lived species will never develop cancer, even when chronically exposed to pollutants or stressful conditions.^36^ Other species are able to thrive with high glucose levels in blood,^37^^,^^38^ when humans would develop diabetes. Interestingly, some hibernating animals display a resistance to muscle atrophy during hibernation.^39^^,^^40^ Understanding the underlying biological mechanisms could help fight cancer, diabetes or human muscular diseases or find nature-inspired treatments, using a mirror research strategy. Animals also display sociality traits and cultural practices that relate to human habits.^41^ Studying social stressors in animal communities, and how the communities overcome stressful conditions, could provide solutions applicable to human societies.

## Tools to study ecosystems exposome

One specificity when studying ecosystems exposome is the sample diversity obtained from the environment. The biotope can provide very tough samples such as rocks and soil, liquid samples as water and gaseous samples as air. The biocenosis also provides diversified samples with specific physico-chemical properties: blood, urine, feces, biopsies, soft and tough tissues (muscle, brain, hair, teeth or bone), microbial colonies or biofilms, leaves, roots, fruits, flowers, and volatiles.

Thanks to recent technological developments and emerging technologies, the tools are now mature to study ecosystems exposome. A discovery-driven approach is allowed thanks to non-targeted analysis based on mass spectrometry.^42^^,^^43^ For example, high-resolution mass spectrometry-based metabolomics can simultaneously provide information on the chemical exposome (pollutants), the metabolome and the lipidome.^27^ From the same sample, the chemical exposure and the biological response can be investigated.^26^

**Figure 3.**
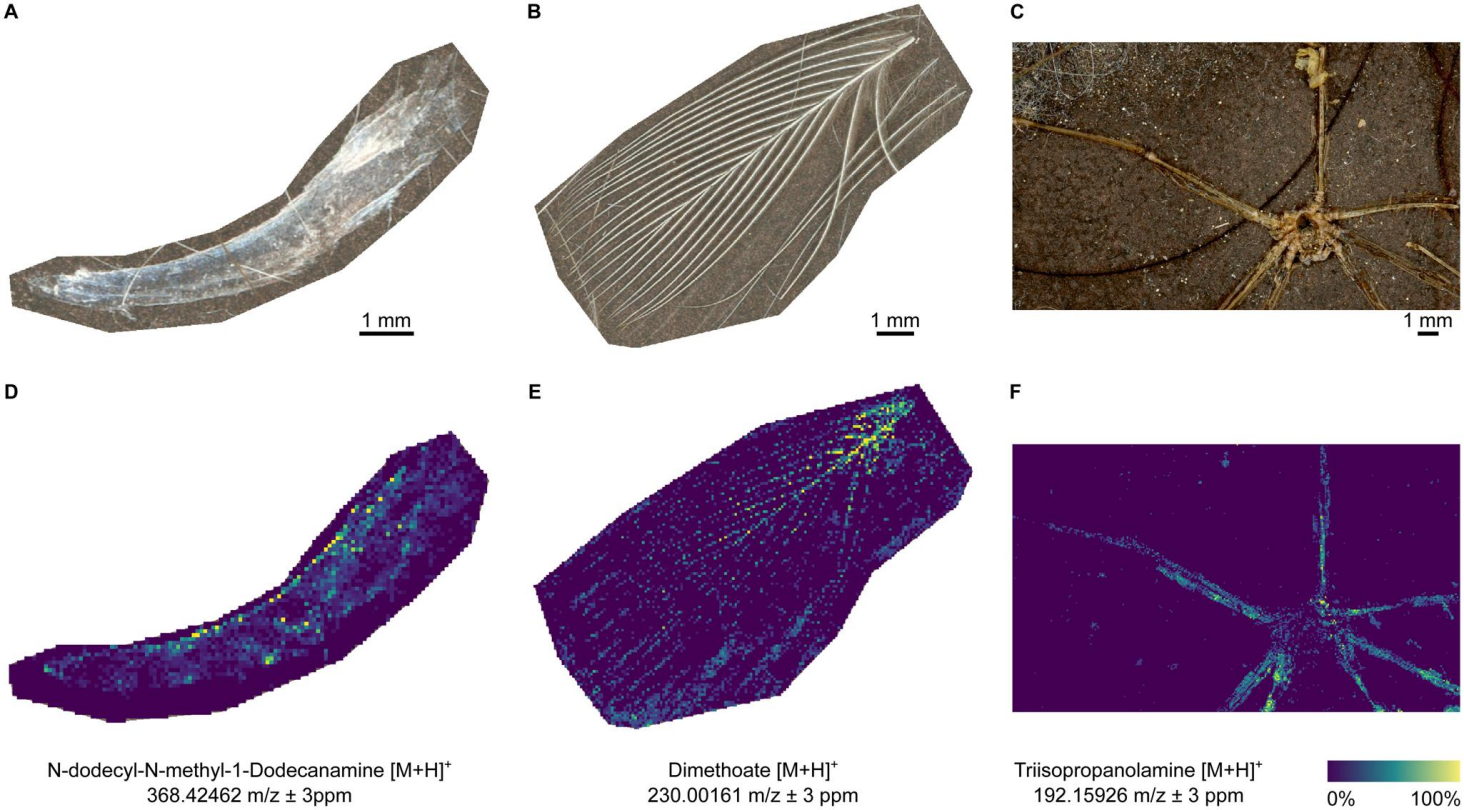
Proof-of-concept use of mass spectrometry imaging to analyze micropollutants in environmental samples. Optical images of cat claw (A), zebra finch fluff (B), and spider full-body recovered from a vacuum cleaner (C). Hazardous chemicals were successfully detected in all the samples as depicted by the molecular images obtained (D–F). Some of these chemicals are of emerging concern, with estrogen and/or androgen receptor activity, carcinogenicity and are irritant for eye and skin, according to the CompTox Chemicals Dashboard (https://comptox.epa.gov/dashboard/). Annotations are at the level 3 of Schymanski classification.^47^

Mass spectrometry imaging (MSI) is increasingly being used to study environmental matrices.^44^ Originally designed to localize compounds (metabolites or proteins) into biological tissue slices, it also demonstrated its ability to analyze more “exotic” samples as bacterial colonies,^45^ paintings^46^ or biosolids.^42^ As a proof-of-concept, we propose MSI as a tool to investigate the exposome of varied animals, with no previous sample preparation (Figure 3). Investigating the chemical load of animals sharing our houses might guide us towards a new vision of indoor exposures. The method also allows the analysis of dust samples, which might be collected from domestic environments to investigate the exposomic load of houses. Although method developments and validations are still needed, MSI is a promising tool to simultaneously co-localize micropollutants and metabolites in environmental samples.

As already used in humans, multi-omics approaches allow the combination of metabolomics, proteomics and transcriptomics or metagenomics.^48^^,^^49^ Plus, ecological sciences can provide a multitude of observations and physiological and behavioral measurements which can be transformed into metadata. These metadata are an incredible source of information to correlate with -omics data in order to better understand ecosystems exposome. The development of artificial intelligence tools will help further investigation of the obtained datasets.^50^ The use of automation and standardization for environmental samples is still to be pushed forward by the exposome community to apply FAIR principles to ecosystems exposome research: Findable, Accessible, Interoperable and Reusable.

## Conclusion

Here, we propose to invite ecosystems into the exposome framework in order to get a wider view of shared exposures, learn from adaptations occurring outside of the human biology, and ecosystems services provided by nature-based solutions. The tools that are developed to study the human exposome could be easily used to study the exposome of ecosystems. Earth (biotope) and its inhabitants (biocenosis, including humans) share the same external components of the exposome across species and ecosystems, which might be called One Exposome as a parallel to the One Health framework. Such a designation may require further discussion of investigators from the exposome and One Health disciplines, to better define the intersections and interconnections between humans and ecosystems in terms of shared general external exposome. As interdisciplinarity is already key in ecosystems sciences (physiology, ethology, and ecology), transdisciplinary collaborations can be proposed between medical doctors, ecologists, biologists, engineers, civil society organizations, institutions or industries. Crossing borders between scientific disciplines but also between science and society could help translate concepts into utility to improve ecosystems and human health.

## Data Availability

Mass spectrometry imaging data presented in Figure 3 are available on MetaSpace (https://metaspace2020.org/project/villette-exposome-2026).
